# Learning to Change

**DOI:** 10.1371/journal.pbio.0020140

**Published:** 2004-05-11

**Authors:** Morten L Kringelbach

## Abstract

A paper published over 20 years ago by Susan Iversen and Mortimer Mishkin on reversal learning continues to inform cognitive neuroscience today

One of the hallmarks of human nature is our remarkably flexible behaviour, especially in the social domain, which is perhaps also a major reason for our relative evolutionary success. Our social skills are already being honed in childhood and early adolescence, when we quickly become very adept at forming and breaking alliances within and between groups and spend much of our time engaged in complex social interactions. At best, these interactions enrich our society; at worst, they become ‘Machiavellian’ and exploitative. While science might appear removed from such politics, many scientists would probably agree that science is in fact a social enterprise, sharing many characteristics with other human pursuits, and that any claim to greater scientific truth can only be accorded over decades, even centuries.

I have always been fascinated by social intelligence, particularly of the ‘Machiavellian’ kind, and found myself wondering at the start of my doctoral research how one might use neuroimaging to study social intelligence in the human brain. I was also interested by the fact that some of this flexible behaviour is shared with other primates such as chimps, bonobos, and even monkeys, who also spend inordinate amounts of time in social interactions, working out social hierarchies. However, it was not immediately obvious how one might go about designing experiments that would address these somewhat intangible issues of social behaviour.

Trawling the scientific literature, I came across the concept of reversal learning. While it is obviously important that we can learn arbitrary associations between stimuli and actions, it is also extremely important that we can relatively easily break these associations and learn others. If we learn that choosing a certain object leads to a reward, it would be rather maladaptive to keep choosing this object when it was no longer associated with a reward but, say, a punishment instead. In order to accommodate complex behaviour, we need to be able to adapt or reverse the learning patterns when things change.

For a long time, it was thought that complex behaviour depended crucially on the prefrontal cortex of the brain, but it was not clear which parts were important for reversal learning. This was investigated in a classic paper by the eminent neuroscientists Susan Iversen and Mortimer Mishkin (1970), who studied lesions in monkeys, with elegant and important results. The authors lesioned discrete parts of the prefrontal cortex in different monkeys and showed convincingly that these lesions had differential effects on the animals' ability to reverse rewarding associations in an object reversal task. When the inferior prefrontal convexity and parts of the lateral orbitofrontal cortex (which is the ventral part of the prefrontal cortex over the orbits) (see Figure 1) were lesioned, the monkeys became significantly impaired with respect to object reversal learning. Specifically, they continued to respond much longer than controls to an object that was no longer rewarded on the first reversal trial.

**Figure 1:**
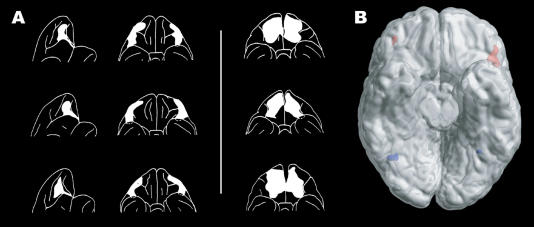
Reversal Learning and the Orbitofrontal Cortex (A) Lateral and ventral views of the surface reconstructions of the lateral and medial orbitofrontal cortex lesions in monkeys (adapted from Iversen and Mishkin 1970), with the former monkeys having difficulty with the reversal task. (B) A ventral view of the human brain, with the cerebellum removed. Red activations in the lateral orbitofrontal cortex indicate the maximal activation for reversal compared to stable acquisition events. Blue activations indicate the main effects of facial expression (adapted from Kringelbach and Rolls 2003).

This was not the case for monkeys who had had the medial parts of the orbitofrontal cortex lesioned. These monkeys were not completely unaffected by the lesion, but showed moderate impairment on all but the first of the object discrimination reversals. Furthermore, they had moderate difficulty withholding response between trials on an auditory differentiation task. These results strongly suggested a differential role for the lateral and medial parts of the orbitofrontal cortex.

Although the paper was not published in a high-profile journal, this elegant and very significant result has had a huge influence on subsequent research. The paper, like many other great papers, was ahead of its time, and it took almost a decade before the citations started to pick up (at last online count, on February 1, 2004, of the ISI database, the paper had generated 229 citations since 1981).

Iversen and Mishkin (1970) persuasively demonstrated the importance of the orbitofrontal cortex in reversal learning, and other studies have since extended this result in nonhuman primates. One study demonstrated that single neurons in the macaque orbitofrontal cortex change their responses to a visual cue after a single trial in which the reward association of the visual cue is reversed (Thorpe et al. 1983). Another lesion study in marmosets by Dias et al. (1996) found that the orbitofrontal cortex is essential for the performance of emotion-related reversal learning tasks.

There was also some evidence that humans with lesions to the orbitofrontal cortex have problems with reversal learning, but the lesions, caused by neurological insult, were not very clean or focal (Rolls et al. 1994). In addition, it had also become clear that lesions to the orbitofrontal cortex were associated with impairments in emotional and social behaviour, characterised by disinhibition, social inappropriateness, and irresponsibility (Anderson et al. 1999).

These interesting but nonconclusive results in humans spurred us on to use neuroimaging on a modified version of a probabilistic reversal learning task designed by Julia Hornak and John O'Doherty (Hornak et al. 2004), whose preliminary data suggested that patients with surgical lesions to the orbitofrontal cortex were impaired. The subjects' task was to determine, by trial and error, which of two stimuli was the more profitable to choose and to keep track of this, reversing their choice when a reversal occurred. By design, the actual reversal event was not easy to determine, since ‘money’ could be won or lost on both stimuli, but a choice of the rewarding stimulus would in general give larger rewards and smaller punishments. The converse was true of the punishing stimulus; losing a large amount of money would often (but not always) signal that a reversal had occurred.

We used functional magnetic resonance imaging to show that dissociable activity in the medial orbitofrontal cortex was correlated with the magnitude of the monetary gains received, while activity in the lateral orbitofrontal cortex was correlated with the monetary losses incurred (O'Doherty et al. 2001). This dissociation between the functions of medial and lateral orbitofrontal cortex seemed to mirror Iversen and Mishkin's initial dissociation in monkeys, in which the lateral orbitofrontal cortex was linked, in both cases, to the reversal trials. However, owing to the probabilistic nature of the task, in which receiving a monetary punishment did not always signal reversal, our imaging study did not reveal the cortical localisation of reversal trials. In addition, our task used money as the secondary reinforcer, which might be a powerful influence on humans but has little biological relevance for other animals, and certainly none in the social domain that I was interested in.

One way to solve these problems was to use facial expressions rather than money as the reinforcing stimuli. This made sense, given that the key to social intelligence is the ability to detect subtle changes in communication and act upon these changes rapidly as they occur. Such changes in social behaviour are often based on facial expression and come so naturally to humans (and are in place so early in child development) that some might argue that this functionality is essentially innate. However, our human social behaviour is sufficiently flexible that we can easily learn to adapt our behaviour to most facial expressions. For example, other people's neutral expressions do not normally indicate that our behaviour should change, but it is easy to think of social contexts in which a neutral expression does indeed imply that our current behaviour is inappropriate and should change.

I designed a reversal task in which the subject's overall goal was to keep track of the mood of two people presented in a pair and, as much as possible, to select the ‘happy’ person, who would then smile. Over time, the person with the ‘happy’ mood (who would smile when selected), changed his/her mood to ‘angry’. This person thus no longer smiled when selected, but instead changed to a facial expression that signalled that he/she should no longer be selected. In the main reversal task, the facial expression used to cue reversal was an angry expression (the most natural facial expression to cue reversal), while in the second, control, version of the reversal task, a neutral expression was used. By using two different reversal tasks in which different facial expressions signalled that behaviour must change, we were able to determine which brain areas were specific to general reversal learning, rather than just to reversal following a particular expression, such as anger.

We used functional magnetic resonance imaging to show that the ability to change behaviour based on facial expression is not reflected in the activity of the fusiform face area (which invariably appears to reflect only identity and not valence), but that general reversal learning is specifically correlated with activity in the lateral orbitofrontal and anterior cingulate/paracingulate cortices (as well as other brain areas, including the ventral striatum and the inferior precentral sulcus) (Kringelbach and Rolls 2003).

This result confirmed and extended the results from Iversen and Mishkin's original paper. Further confirmation came from the neuropsychological testing, carried out by Julia Hornak on human patients with surgical lesions to the orbitofrontal cortex, which showed that bilateral (but not unilateral) lesions to the lateral orbitofrontal cortex produce significant impairments in reversal learning (Hornak et al. 2004). Yet, as always, these results are not conclusive and raise many new issues. It is, for instance, not presently clear what other areas of the brain are necessary and sufficient for reversal learning. Among the other brain areas we found relating to general reversal learning in our study, the ventral striatum is, for instance, an obvious candidate (Cools et al. 2002). In addition, functional magnetic resonance imaging is essentially a correlative technique, with poor temporal information, which makes it very difficult to infer causal relations between brain regions. Thus, further investigations, e.g., with magnetoencephalography, will still be required to gain temporal information on the milliseconds scale. I take heart from a friend, a very distinguished scientist, who states that the price for having spent a lifetime in cutting-edge research is that 99% of his (and other scientists') research is wrong—perhaps not completely wrong, but certainly wrong in the details. I would like to think that the original result from the Iversen and Mishkin paper is among the rare 1%, but the trouble with such foresight is that it lacks the vantage point of true hindsight.

In his masterpiece, *The Prince*, Niccolò Machiavelli offers a rather pessimistic view on human nature, in which ‘love is held by a chain of obligation which, since men are bad, is broken at every opportunity for personal gain’. It may be that our capacity for rapid reversal learning is sometimes used for less than noble pursuits, both in science and in interpersonal relations in general, but we would be in real trouble if we couldn't learn to change.
